# Proteomic Analysis Reveals Changes in Tight Junctions in the Small Intestinal Epithelium of Mice Fed a High-Fat Diet

**DOI:** 10.3390/nu15061473

**Published:** 2023-03-19

**Authors:** Hisanori Muto, Takashi Honda, Taku Tanaka, Shinya Yokoyama, Kenta Yamamoto, Takanori Ito, Norihiro Imai, Yoji Ishizu, Keiko Maeda, Tetsuya Ishikawa, Shungo Adachi, Chikara Sato, Noriko M. Tsuji, Masatoshi Ishigami, Mitsuhiro Fujishiro, Hiroki Kawashima

**Affiliations:** 1 Department of Gastroenterology and Hepatology, Nagoya University Graduate School of Medicine, 65 Tsuruma-cho, Showa-ku, Nagoya 466-8550, Japan; 2 Biological Systems Control Team, Biomedicinal Information Research Center, National Institute of Advanced Industrial Science and Technology (AIST), 2-3-26 Aomi, Koto-ku, Tokyo 135-0064, Japan; 3 School of Integrative and Global Majors (SIGMA), Tsukuba University, 1-1-1 Tennodai, Tsukuba 305-8577, Japan; 4 Biological Science Course, Graduate School of Science and Engineering, Aoyama Gakuin University, 5-10-1 Fuchinobe, Chuou-ku, Sagamihara 252-5258, Japan; 5 Division of Immune Homeostasis, Department of Pathology and Microbiology, Nihon University School of Medicine, 30-1 Oyaguchi-Kamimachi, Itabashi, Tokyo 173-8610, Japan; 6 Division of Microbiology, Department of Pathology and Microbiology, Nihon University School of Medicine, 30-1 Oyaguchi-Kamimachi, Itabashi, Tokyo 173-8610, Japan; 7 Division of Cellular and Molecular Engineering, Department of Life Technology and Science, National Institute of Advanced Industrial Science and Technology (AIST), 1-1-1 Higashi, Tsukuba 305-8560, Japan; 8 Microbiology and Immunology, School of Dentistry at Matsudo, Nihon University, 22-870-1 Sakae-cho-nishi, Tokyo 271-8587, Japan; 9 Department of Food Science, Jumonji University, 2-1-28 Sugasawa, Niiza 352-8510, Japan; 10 Department of Gastroenterology, Graduate School of Medicine, The University of Tokyo, 7-3-1, Hongo, Bunkyo-ku, Tokyo 113-0033, Japan

**Keywords:** non-alcoholic fatty liver disease, intestinal epithelial cells, proteomic analysis, tight junctions, Cldn 7, Epcam, upper small intestine

## Abstract

The impact of a high-fat diet (HFD) on intestinal permeability has been well established. When bacteria and their metabolites from the intestinal tract flow into the portal vein, inflammation in the liver is triggered. However, the exact mechanism behind the development of a leaky gut caused by an HFD is unclear. In this study, we investigated the mechanism underlying the leaky gut related to an HFD. C57BL/6J mice were fed an HFD or control diet for 24 weeks, and their small intestine epithelial cells (IECs) were analyzed using deep quantitative proteomics. A significant increase in fat accumulation in the liver and a trend toward increased intestinal permeability were observed in the HFD group compared to the control group. Proteomics analysis of the upper small intestine epithelial cells identified 3684 proteins, of which 1032 were differentially expressed proteins (DEPs). Functional analysis of DEPs showed significant enrichment of proteins related to endocytosis, protein transport, and tight junctions (TJ). Expression of Cldn7 was inversely correlated with intestinal barrier function and strongly correlated with that of Epcam. This study will make important foundational contributions by providing a comprehensive depiction of protein expression in IECs affected by HFD, including an indication that the Epcam/Cldn7 complex plays a role in leaky gut.

## 1. Introduction

The prevalence of non-alcoholic fatty liver disease (NAFLD) has been increasing worldwide in recent years and is a major public concern [1]. Even in Japan, where obesity is less common than in Western countries, the prevalence of NAFLD is expected to increase in the future [2,3]. Among NAFLD patients, 12–40% develop non-alcoholic steatohepatitis (NASH), which can progress to cirrhosis and hepatocellular carcinoma [4]. During the progression of NASH, the multiple hit theory proposes that obesity and insulin resistance are the first hits, followed by increased oxidative stress, adipokine abnormalities, endotoxin, and other factors, leading to inflammation, which in turn promotes fibrosis and the activation of hepatic stellate cells [5].

Recently, abnormalities in the gut microbiota and the resulting increased intestinal permeability have been shown to promote the progression of NASH [6]. The gut-liver axis, which refers to the interdependent relationship between the gut, microbiota, and liver, is implicated in this process [7]. Increased permeability of the upper small intestine and tight junction (TJ) dysfunction has been reported in patients with NAFLD [8,9]. Dysfunctions of the intestinal barrier, including the TJ, were shown to be an important etiology of NASH progression in mouse studies [10]. This disruption of the TJ is thought to result from changes in the microbiota, including small intestinal bacterial overgrowth [11,12,13]. A high-fat diet (HFD) alters the microbiota, which compromises the intestinal barrier and promotes the influx of bacterial products into the portal vein [10]. Studies in rodents have shown that an HFD modifies the composition of the intestinal microbiota, which subsequently exacerbates intestinal barrier dysfunction and stimulates hepatic inflammation [14,15]. Because the liver is exposed to high concentrations of pathogen-associated molecular patterns, which are molecular motifs commonly found in microorganisms that promote inflammation, it is particularly vulnerable to their effects, particularly when primed by subclinical pathologies, such as the accumulation of lipids within hepatocytes [7]. Although recent studies have often attempted to ameliorate NASH by supplementation with prebiotics and probiotics to restore the intestinal bacterial balance caused by HFD [16,17,18], few studies have focused on changes in the host intestinal TJ itself due to HFD ingestion. Detailed study of changes in the host intestinal TJ induced by HFD ingestion may lead to the development of new biomarkers and therapies for the progression of NASH.

Because TJ functions are regulated by vesicular trafficking and redistribution of its component proteins [19], transcriptome analysis may not be sufficient to investigate its effects. Deep proteomics using mass spectrometry detects and quantifies thousands of proteins in a single experiment, providing a comprehensive view of the proteome. This approach is suitable for studying complex biological processes, such as signaling pathways and protein-protein interactions, and for identifying novel biomarkers for disease diagnosis and treatment [20]. Therefore, we used high-throughput deep proteomic analysis to comprehensively analyze the protein expression profiles of mouse intestinal epithelial cells (IECs) fed an HFD to investigate the mechanism of TJ dysfunction.

## 2. Materials and Methods

### 2.1. Murine Model

Nine-week-old male C57BL/6J mice from Japan SLC (Shizuoka, Japan) were fed an HFD containing 60 kcal% fat (D12492; Research Diet, New Brunswick, NJ, USA) or a control diet (CD, D12450J; Research Diet) for 24 weeks. Mice were housed with free access to water and food at a temperature of 23 ± 1 °C, a humidity of 50 ± 10%, and a 12-h light/dark cycle. After the animals were fully anesthetized and sacrificed, the liver, small intestine, and serum were collected for histological and serological analyses. Five mice were included in the HFD group and four mice in the CD group. Animal experiments were conducted in accordance with the NIH Guidelines for the Care and Use of Laboratory Animals.

### 2.2. Intestinal Permeability

Intestinal permeability was assessed using previously described methods [21]. Briefly, 4-kDa fluorescein isothiocyanate (FITC)-dextran (Sigma-Aldrich, St. Louis, MO, USA; 20 mg/mL, PBS) was administered orally to mice after a 4-h fast (9:00 to 13:00) at a dose of 200 mg/kg body weight. Four hours later, blood was collected from the retrobulbar capillary plexus into heparinized tubes. Plasma was obtained by centrifugation at 2000× *g* for 5 min, followed by a 1:5 (*v*/*v*) dilution in PBS. Spectrophotometric measurement of fluorescence was performed using a SpectraMax iD5 (Molecular Devices, Tokyo, Japan) and a 96-well plate (excitation: 485 nm, emission: 528 nm) to determine the concentration of FITC-dextran.

### 2.3. Histological and Immunohistochemical Analyses

Liver and intestine samples were fixed in 4% paraformaldehyde, paraffin-embedded, sectioned at 4 μm thickness, and stained with hematoxylin and eosin (H&E). Tissue images were captured using a BZ-X800 microscope (Keyence, Osaka, Japan). The NAFLD activity score (NAS) was used to assess the activity and severity of fatty liver. The NAS is calculated based on three histologic features in the liver: steatosis (0–3), hepatocellular ballooning (0–2), and lobular inflammation (0–3) [22]. The lipid droplet area was calculated using a BZ-X800 analyzer (Keyence). For immunohistochemical analysis, 4 μm paraffin-embedded tissue sections were deparaffinized, rehydrated, and subjected to antigen retrieval by heating in preheated 10 mM sodium citrate (pH 6.0) at 98 °C for 20 min. The sections were blocked with 5% (vol/vol) bovine serum albumin for 30 min and then incubated overnight with primary antibodies to either anti-zonula occludens-1 (ZO-1) (Abcam, Cambridge, UK) or anti-Epcam (rabbit IgG, Abcam). Anti-rabbit IgG Alexa Fluor 488 was used as the secondary antibody and counterstained with 4′,6-diamidino-2-phenylindole (DAPI; Sigma-Aldrich). Tissue images were captured with a BZ-X800 microscope.

### 2.4. Isolation of Intestinal Epithelial Cells

The small intestine was divided into two equal parts: the upper small intestine and the lower small intestine. Isolation of IECs was performed as described previously [23]. Briefly, each piece was washed with PBS and cut into 1–2 cm pieces. The sections were incubated in isolation buffer (20 mM HEPES, 10 mM EDTA, 1 mM sodium pyruvate, 10% FCS, and 1% penicillin-streptomycin in PBS) at 37 °C for 25 min. After passing through a 100-μm cell strainer, the samples were centrifuged at 3500 rpm for 5 min, washed with PBS, and stored at −80 °C for protein mass spectrometry analysis.

### 2.5. Proteomic Assay

The proteomic analysis technique was performed according to previously published methods [24]. For the sample used in mass spectrometry analysis, we made slight modifications to the Mass Spec Sample Prep Kit for Cultured Cells (Thermo Fischer Scientific, Waltham, MA, USA) protocol. Briefly, we suspended the pelletized cells in a lysis buffer, adding benzonase to degrade nucleic acids, and precipitating proteins using acetone. The precipitated protein was then re-dissolved in guanidine hydrochloride, reduced with TCEP, alkylated with iodoacetamide, and digested with lysyl endopeptidase and trypsin. The resulting digested peptides were analyzed using an Evosep One LC system (Evosep biosystems, Odense, Denmark) connected to a Q-Exactive HF-X mass spectrometer (Thermo Fischer Scientific). The mobile phases consisted of 0.1% formic acid as solution A and 0.1% formic acid/99.9% Acetonitrile as solution B. The analysis was performed in data-dependent acquisition mode, and the top 25 recorded mass spectrometry spectra between 380 and 1500 m/z were selected. All MS/MS spectra were searched against the protein sequences of the mouse Swiss-Prot database using Proteome Discoverer 2.2 with the SEQUEST search engine. The false discovery rate (FDR) was set to 1% on peptide spectrum match.

### 2.6. Statistical Analysis

The proteomic data obtained from the intestinal epithelial cells, which involved a comparison between two groups, were analyzed using the online statistical software MetaboAnalyst version 5.0 (https://www.metaboanalyst.ca/ accessed on 11 December 11) [25]. Functional analyses of the differentially expressed proteins (DEPs) were performed using DAVID (Database for Annotation Visualization and Integrated Discovery) version 2021 (https://david.ncifcrf.gov/ accessed on 11 December 2022) [26,27]. Kyoto Encyclopedia of Genes and Genomes (KEGG) pathway enrichment analysis and gene ontology (GO) functional annotation clustering were performed using DAVID. The software MetaboAnalyst was employed to identify DEPs and conduct Principal Component Analysis (PCA) utilizing the default settings. In instances of missing data, a value of 1/5 of the minimum positive value for each variable was assigned. Multiple comparisons were performed, and proteins with an FDR value of less than 0.05 were considered DEPs. The DEPs were then subjected to KEGG analysis and functional annotation clustering using the DAVID. Other statistical analyses were performed using Graph Pad Prism version 9.2.0 (GraphPad Software, San Diego, CA, USA). Data were presented as bar graphs showing the mean ± standard error of the mean. Student’s *t*-test or Mann-Whitney U test was used for continuous variables to compare the two groups, as appropriate. A *p*-value < 0.05 was considered statistically significant.

## 3. Results

### 3.1. Evaluation of the Liver and Changes in Intestinal Permeability Induced by an HFD in Mice

**Figure 1:**
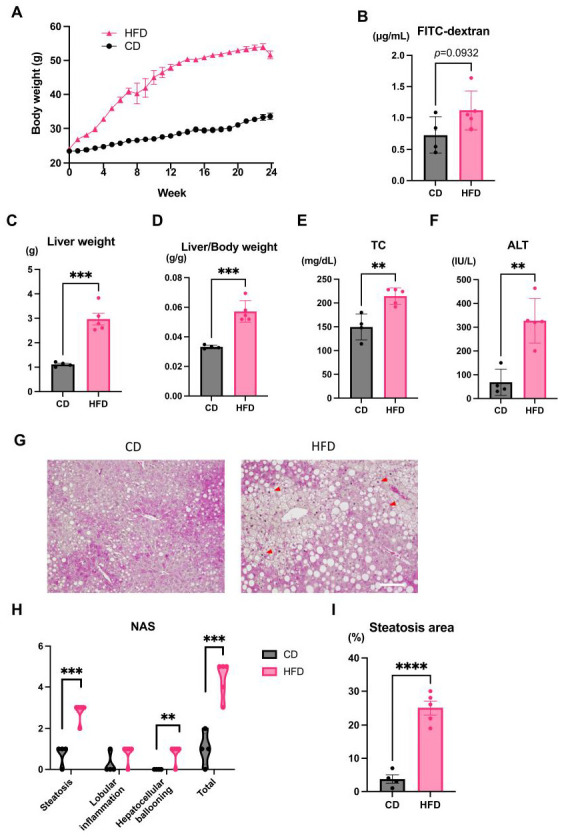
Mouse model of high-fat diet (HFD) induced nonalcoholic fatty liver. (**A**) Body weight gain of HFD- and control diet (CD)-fed mice. (**B**) The concentration of blood fluorescein isothiocyanate (FITC)-dextran following oral administration to mice. (**C**–**F**) Liver weight, Liver/body weight ratio, serum total cholesterol (TC), and serum aspartate aminotransferase (ALT) of the HFD and CD groups. (**G**) Hematoxylin and eosin (H&E) staining of livers from HFD- and CD-fed mice. Scale bar: 100 μm. Red Arrowheads show ballooning cells. (**H**) The non-alcoholic fatty liver disease activity score (NAS) is the sum of the lobular inflammation, ballooning, and steatosis scores according to the H&E score. (**I**) Area of liver steatosis of HFD- and CD-fed mice. All data represent the mean ± standard error of the mean. Student’s *t*-test, ** *p* < 0.01, *** *p* < 0.001, **** *p* < 0.0001. *n* = 4, CD group; *n* = 5 HFD group.

To induce hepatic steatosis, mice were fed an HFD for 24 weeks, and a control group was maintained on a CD. The body weight of mice in the HFD group was significantly higher than that in the CD group (Figure 1A). An in vivo intestinal permeability assay showed a trend toward increased intestinal permeability in the HFD group mice compared with CD-fed mice (Figure 1B). All mice were sacrificed after 24 weeks of HFD. The liver weight and liver/volume ratio of the HFD group were significantly higher than those of the CD group (Figure 1C,D). The HFD group also showed elevated serum alanine transaminase (ALT) and total cholesterol (TC) serum levels compared with the CD group (Figure 1E,F).

**Figure 2:**
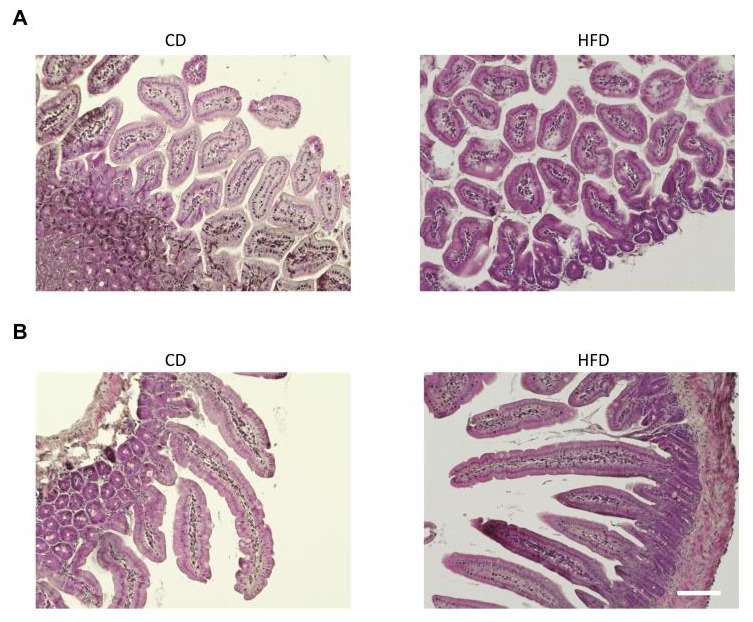
H&E staining of the upper (**A**) and lower (**B**) small intestines from HFD- and CD-fed mice. Scale bar: 100 μm.

**Figure 3:**
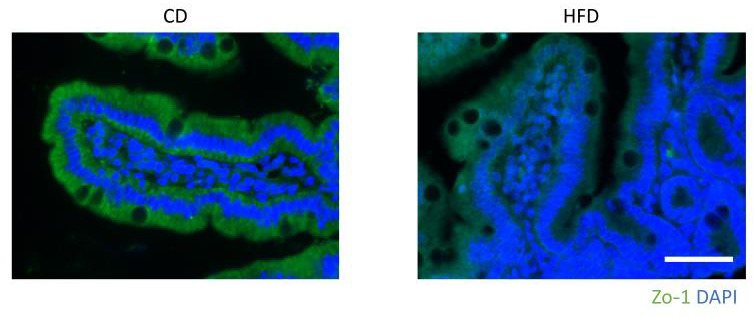
Representative immunohistological ZO-1 stained images of the upper small intestine of HFD- and CD-fed mice. Scale bar: 50 μm.

H&E staining of the liver showed hepatic steatosis and mild hepatocyte ballooning degeneration in HFD-fed mice and a significant increase in NAFLD activity score (Figure 1G–I). The upper small intestine and lower small intestine showed no obvious differences in H&E staining (Figure 2A,B). Fluorescence immunostaining showed that the localization of Zo-1 on the plasma membrane of IECs in the upper small intestine was decreased in the HFD group, suggesting TJ dysfunction (Figure 3).

### 3.2. Proteomic Changes in the Upper and Lower Small Intestines Induced by an HFD

**Figure 4:**
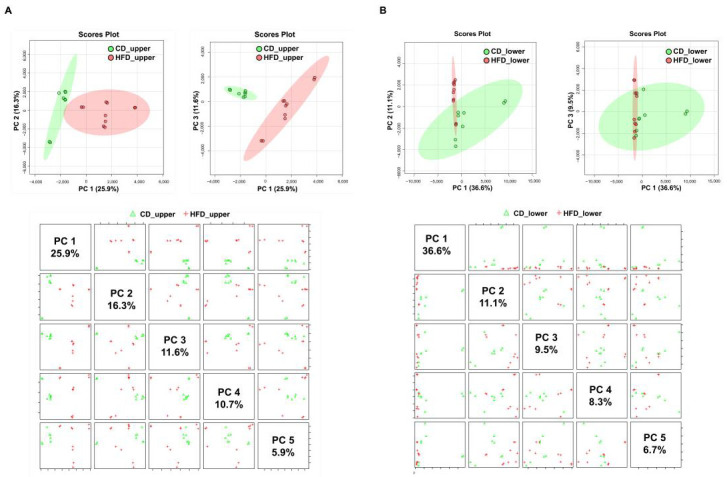
High-throughput deep proteomics of intestinal epithelial cells (IECs) in the upper and lower small intestines. (**A**) Principal component analysis (PCA) for proteomes of IECs from the upper small intestine. (**B**) PCA for proteomes of IECs from the lower small intestine. Green and red areas show 95% confidence regions.

Proteomic changes in the IECs of the upper and lower small intestines of mice were analyzed by quantitative analysis using LC-MS/MS. Proteins in the upper and lower small intestinal IECs of the CD and HFD groups were analyzed twice per sample. PCA of the protein expression profiles by proteome analysis revealed that in IECs of the upper small intestine, the global pattern of the proteome was clearly different between the HFD and CD groups (Figure 4A). In the lower small intestine, there was no obvious difference in the global pattern between the HFD and CD groups (Figure 4B). In addition, the global pattern of PCA in the proteome of the upper and lower small intestines of the HFD group was clearly different (Figure S1). Because HFD-induced changes were more pronounced in the upper small intestines, the following analyses were performed in the upper small intestines.

### 3.3. Gene Ontology and KEGG Pathway Enrichment Analysis of Differentially Expressed Proteins in the Upper Small Intestine

**Figure 5:**
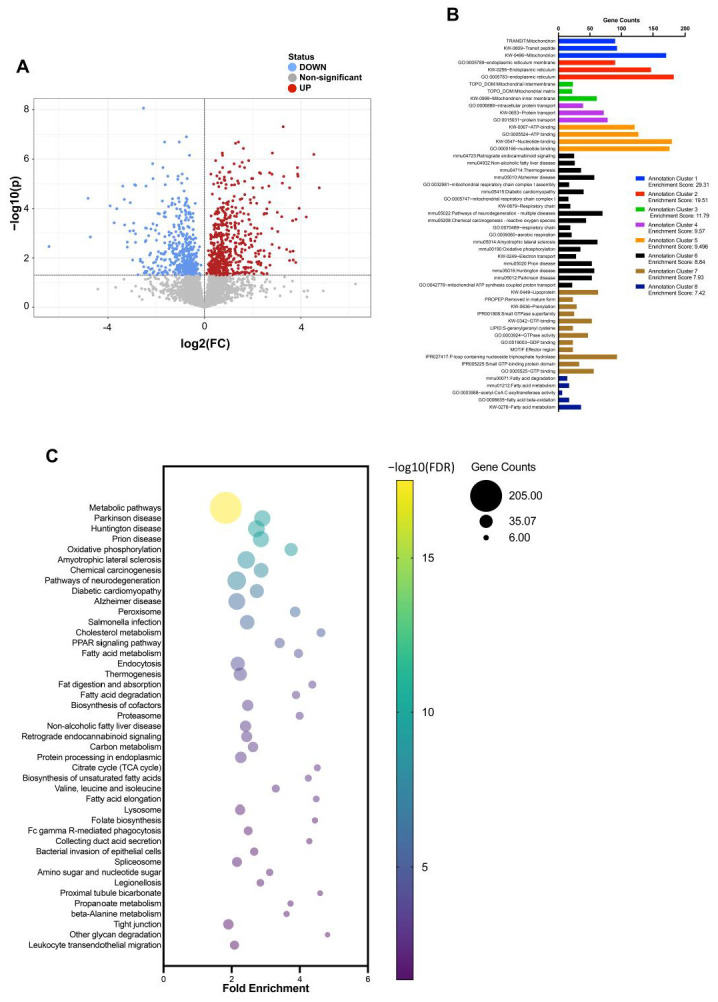
High-throughput deep proteomics of IECs from the upper small intestine of HFD- and CD-fed mice. (**A**) Volcano plot of differentially expressed proteins (DEPs). Red and blue dots represent upregulated and downregulated proteins, respectively. (**B**) Functional annotation clustering of DEPs by DAVID. (**C**) Kyoto Encyclopedia of Genes and Genomes (KEGG) pathway enrichment of DEPs.

**Figure 6:**
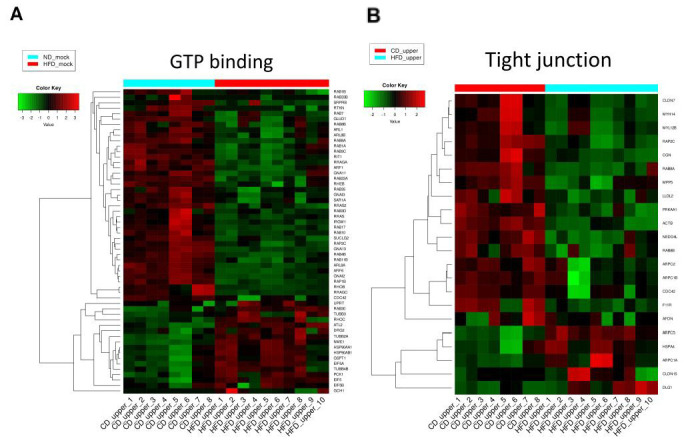
Heatmaps of DEPs include “GTP binding” (GO: 0005525, (**A**)) and “Tight Junction” (mmu 04530, (**B**)).

Overall, 3684 proteins were identified in IECs from the upper small intestine, and 1032 proteins showed significant differences in expression between the HFD and CD groups (FDR < 0.05) (Figure 5A). Further bioinformatic analysis was performed on the DEPs identified. Functional annotation clustering by DAVID revealed that functional clusters related to mitochondria, endoplasmic reticulum, protein transport, and GTP-binding proteins were enriched (Figure 5B). Fifty-six of the GTP binding (GO:0005525) proteins were identified as DEPs and contained several Rab family proteins (Figure 6A), which regulate intracellular membrane trafficking [28]. Many Rab family proteins, such as Rab4b, Rab7, Rab11b, and Rab35, were downregulated in the HFD group compared with the CD group (Figure 6A). In the KEGG pathway enrichment analysis, metabolic pathways, the PPAR pathway, and endocytosis were enriched, and TJ was also significantly enriched (Figure 5C). Twenty-two proteins in TJ (mmu 04530) were identified as DEPs (Figure 6B).

### 3.4. Correlations of TJ Proteins and Intestinal Permeability

**Figure 7:**
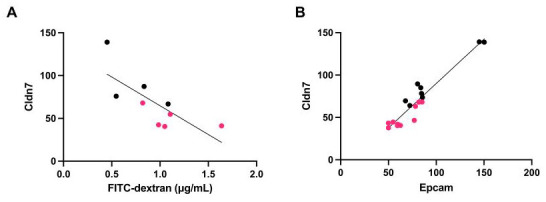
Correlations of claudin (Cldn)7 with intestinal permeability (**A**) and Epcam (**B**). Red and black dots indicate samples from HFD- and CD-fed mice, respectively (A; *n* = 4, CD group; *n* = 5 HFD group, B; *n* = 8, CD group; *n* = 10).

We then investigated the correlation between 22 TJ-associated proteins and intestinal permeability. The correlation between intestinal permeability in the in vivo FITC-dextran assay and protein expression quantified by LC-MS/MS is shown in Table 1. Significant inverse correlations were found for nine proteins, and interestingly, relatively strong correlations were found with actin-myosin fiber expression. Claudin-7 (Cldn7), a major membrane protein involved in TJ formation, also showed a significant correlation with intestinal permeability (Figure 7A).

**Figure 8:**
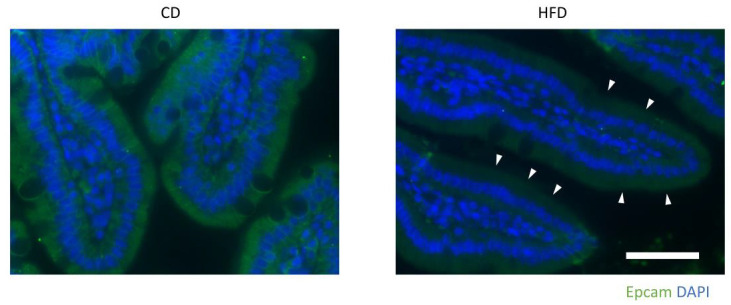
Representative immunohistological Epcam stained images of the upper small intestines from HFD- and CD-fed mice. Scale bar: 50 μm. Arrowheads indicate decreased Epcam signal at the plasma membrane of IECs of HFD-fed mice.

Based on the quantitative proteomics results, we searched for proteins that were strongly correlated with Cldn7 (Figure S2). Among these proteins, the expression levels of Cldn7 and Epcam showed a very strong correlation (Figure 7B), as both are known to interact with each other to regulate TJ functions [29,30]. Indeed, fluorescence immunostaining showed that the Epcam signal was reduced in the HFD group compared with the CD group (Figure 8).

## 4. Discussion

In the present study, the protein expressions of IECs from the upper and lower small intestine of mice fed an HFD, or a CD were comprehensively analyzed by high-throughput deep proteome analysis to investigate the mechanism of TJ dysfunction. We found that HFD, especially in the upper small intestine, alters the protein expression profile of IECs and results in the dysregulation of several pathways related to protein transport, including endocytosis. In addition, several TJ-related proteins were differentially expressed in IECs of mice fed HFD compared to a normal diet.

The TJs are complex protein structures that hold adjacent cells together, creating a barrier that regulates the passage of substances through the intestinal mucosa [31]. When the TJs become compromised, they can lead to intestinal permeability, also known as “leaky gut” syndrome [32]. The interaction between the intestine and the liver is mediated by the portal vein, which can transfer nutrients, as well as products of intestinal origin, such as microbial metabolites and microbial components, to the liver. This enterohepatic circulation provides the liver with continuous exposure to gut-derived factors. Furthermore, this gut-liver relationship is referred to as the “gut-liver axis”, and its importance in liver homeostasis and disease pathogenesis is being increasingly recognized [33]. TJ dysfunction has been observed in obese patients [34] and patients with NASH [9]. Intestinal barrier dysfunction is one of the main causes of NASH progression [7,35,36] and is an emerging therapeutic target [6,16]. Although HFD is known to damage the intestinal barrier directly or indirectly, with particular emphasis on the involvement of the microbiota [37], few reports have comprehensively investigated changes in host IECs. Therefore, the present data should be very valuable in elucidating the mechanism of HFD-induced intestinal barrier dysfunction for the following reasons.

The results of this study revealed three important points. First, highly sensitive quantitative deep proteomics was a very valuable tool for the comprehensive analysis of TJ and related proteins expressed in IECs. Because the localization of TJ proteins is not necessarily dependent on transcription, it may be insufficient to assess their changes by messenger RNA expression analysis [38]. The structure of the TJ is composed of transmembrane adhesion proteins, cytoplasmic plaque proteins, and the actin cytoskeleton. Recent studies suggest that the TJ is not as rigid as previously thought but is a highly dynamic structure that can respond to various biochemical and mechanical stimuli by reforming and remodeling [39]. The dynamics of TJ proteins are mainly regulated by endocytosis [40]. It is highly suggestive that deep proteomics in this study revealed that HFD-induced changes in TJ proteins in mouse IECs occurred together with proteins associated with endocytosis and protein trafficking and their regulatory GTP-binding proteins. One potential pathway of endocytic sorting is recycling [41]. GTP-binding proteins that were decreased in the HFD group, Rab4, Rab7, Rab11, and Rab35, were reported to be associated with recycling endosomes [41], and dysfunction of this pathway may have affected TJ formation.

Second, we found that the changes in the protein expression of IECs induced by an HFD differed significantly between the upper and lower small intestines. Indeed, HFD-induced changes in the global pattern of protein expression were more pronounced in the upper small intestine. Furthermore, we have shown that Cldn7 is significantly downregulated by HFD in upper small intestinal IECs, which correlates with intestinal barrier dysfunction. In our model, this suggests that the changes in the upper small intestine have a strong impact on intestinal barrier function. The region of the intestine where leaky gut occurs in the development of NASH remains unclear. Future studies of NASH focusing on intestinal barrier dysfunction should examine differences by the intestinal site. Although our data suggest that the HFD-induced intestinal barrier dysfunction is more pronounced in the upper small intestine, in clinical situations, the lower small intestine and colon may also be relevant because obesity, other habits, and genetic factors, as well as HFD, are intricately related to the progression of NASH [42].

Finally, we found that Epcam-mediated regulation of Cldn7 and its associate protein Epcam may play important roles in HFD-induced TJ dysfunction. Cldn7 is a transmembrane protein that plays an important role in maintaining TJ integrity and permeability but is characterized by a stronger basement membrane distribution than other claudins and has been shown to have functions in maintaining epithelial cell-matrix interactions and intestinal homeostasis [29]. Cldn7 is also known to interact with Epcam, a glycoprotein expressed in some epithelia, and regulate TJ functions in the intestine. Epcam interacts directly with Cldn7 at the lateral basement membrane and TJs of the intestinal epithelium, regulating the organization and function of the TJs [43,44]. A complex downregulation of claudin was observed in the intestinal epithelium of Epcam mutant mice, indicating that intestinal TJs were affected and intestinal permeability was increased [45,46]. Consistently, Epcam knockdown has also been shown to cause TJ dysfunction in the T84 and Caco-2 cell lines [47].

Here the expression levels of Cldn7 and Epcam showed a strong correlation, and Epcam-mediated regulation of Cldn7 seems to be associated with HFD-induced TJ dysfunction. The relationship between Epcam/Cldn7 interaction and protein trafficking, including endocytosis, has not been previously reported and requires further investigation. Nevertheless, the comprehensive protein expression analysis provides fundamental data to elucidate the functional relevance of endocytosis and GTP-binding proteins in HFD-induced intestinal barrier dysfunction and to elucidate the pathogenesis of NASH. Although additional mechanistic investigations and validation in humans are needed, these data may prove valuable in validating novel key factors and therapeutic approaches for NASH.

This study has several limitations. First, the present study establishes a correlation between the expression of Cldn7 and the function of the intestinal barrier, but it does not prove a causal relationship. This study focused only on small intestinal IECs and did not investigate other mechanisms or factors that may contribute to HFD-induced leaky gut. Further studies are needed to fully understand the specific role of Cldn7 in the pathogenesis of leaky gut and the underlying mechanisms of TJ dysfunction. Second, this study did not examine the effects of different types of dietary fat or the duration of HFD on intestinal permeability. These factors may also contribute to the development of leaky gut and should be investigated in future studies. Third, this study did not investigate the role of the gut microbiota in relation to comprehensive protein expression in IECs. Further research is necessary to understand the potential influence of the gut microbiota on the development of leaky gut and its association with Cldn7 expression. Finally, because the study was conducted in C57BL/6J mice, the findings may not be directly applicable to humans. Further validation in humans is needed.

## 5. Conclusions

We investigated protein changes associated with TJ by the comprehensive analysis of protein expression in IECs from HFD-fed mice using highly sensitive deep proteomics. Deep proteomic analysis is a powerful tool for investigating the underlying molecular mechanisms of NASH. This study provides new insights into the role of TJ dysfunction in the pathogenesis of these diseases and identifies potential candidate biomarkers for their diagnosis and treatment. We showed that Cldn7/Epcam in upper small intestinal IECs might be one of the key factors and a potential biomarker. Further studies are needed to validate these findings in humans and to develop targeted therapies based on these biomarkers.

## Figures and Tables

**Table 1:** Correlations of TJ-related proteins and intestinal permeability.

Protein	Pearson r	95% Confidence Interval	R Squared	*p* (Two-Tailed)	*p* Value Summary
*F11r*	−0.1358	−0.7338 to 0.5807	0.01845	0.7275	ns
*Llgl2*	−0.4785	−0.8671 to 0.2720	0.229	0.1925	ns
*Rab8a*	−0.7657	−0.9478 to −0.2066	0.5862	0.0162	*
*Rab8b*	−0.447	−0.8568 to 0.3088	0.1998	0.2277	ns
*Rap2c*	−0.7321	−0.9395 to −0.1324	0.536	0.0249	*
*Arpc1a*	0.8983	0.5805 to 0.9786	0.8069	0.001	***
*Arpc1b*	−0.1386	−0.7351 to 0.5788	0.01921	0.7221	ns
*Arpc2*	−0.07812	−0.7056 to 0.6181	0.006102	0.8417	ns
*Arpc5*	0.7178	0.1025 to 0.9358	0.5152	0.0295	*
*Actb*	−0.4112	−0.8446 to 0.3480	0.1691	0.2716	ns
*Afdn*	−0.1252	−0.8026 to 0.6932	0.01568	0.7891	ns
*Cdc42*	−0.009934	−0.6696 to 0.6585	9.87 × 10^−5^	0.9798	ns
*Cgn*	−0.692	−0.9291 to −0.05163	0.4789	0.0389	*
*Cldn15*	−0.0411	−0.7703 to 0.7347	0.001689	0.9303	ns
*Cldn7*	−0.7494	−0.9438 to −0.1698	0.5616	0.0201	*
*Dlg1*	0.1179	−0.5926 to 0.7252	0.0139	0.7626	ns
*Hspa4*	0.8191	0.3399 to 0.9606	0.6709	0.0069	**
*Myh14*	−0.8424	−0.9660 to −0.4046	0.7096	0.0044	**
*Myl12b*	−0.8336	−0.9640 to −0.3797	0.6949	0.0052	**
*Nedd4l*	−0.4019	−0.8414 to 0.3577	0.1615	0.2836	ns
*Mpp5*	−0.7453	−0.9507 to −0.08549	0.5554	0.0338	*
*Prkaa1*	−0.4497	−0.8577 to 0.3057	0.2022	0.2246	ns

ns: not significant, * *p* < 0.05, ** *p* < 0.01, *** *p* < 0.001.

## Data Availability

The data presented in this study are openly available in the NAGOYA repository (DOI: 10.18999/2004734, accessed on 7 February 2023).

## Supplementary Materials

The following supporting information can be downloaded at: https://www.mdpi.com/article/10.3390/nu15061473/s1, Figure S1: Proteomes of IECs from HFD- or CD-fed mice comparing upper and lower small intestines.; Figure S2: Proteins strongly correlated with Cldn7.
