# Supplementary material for: Proteomic Analysis Reveals Changes in Tight Junctions in the Small Intestinal Epithelium of Mice Fed a High-Fat Diet
Source: Nutrients. 2023 Mar 19;15(6):1473. doi: 10.3390/nu15061473 (PMC10056729; doi:10.3390/nu15061473)
Supplement: Supplementary file 1 [file nutrients-15-01473-s001.zip › nutrients-2258935-supplementary.pdf]

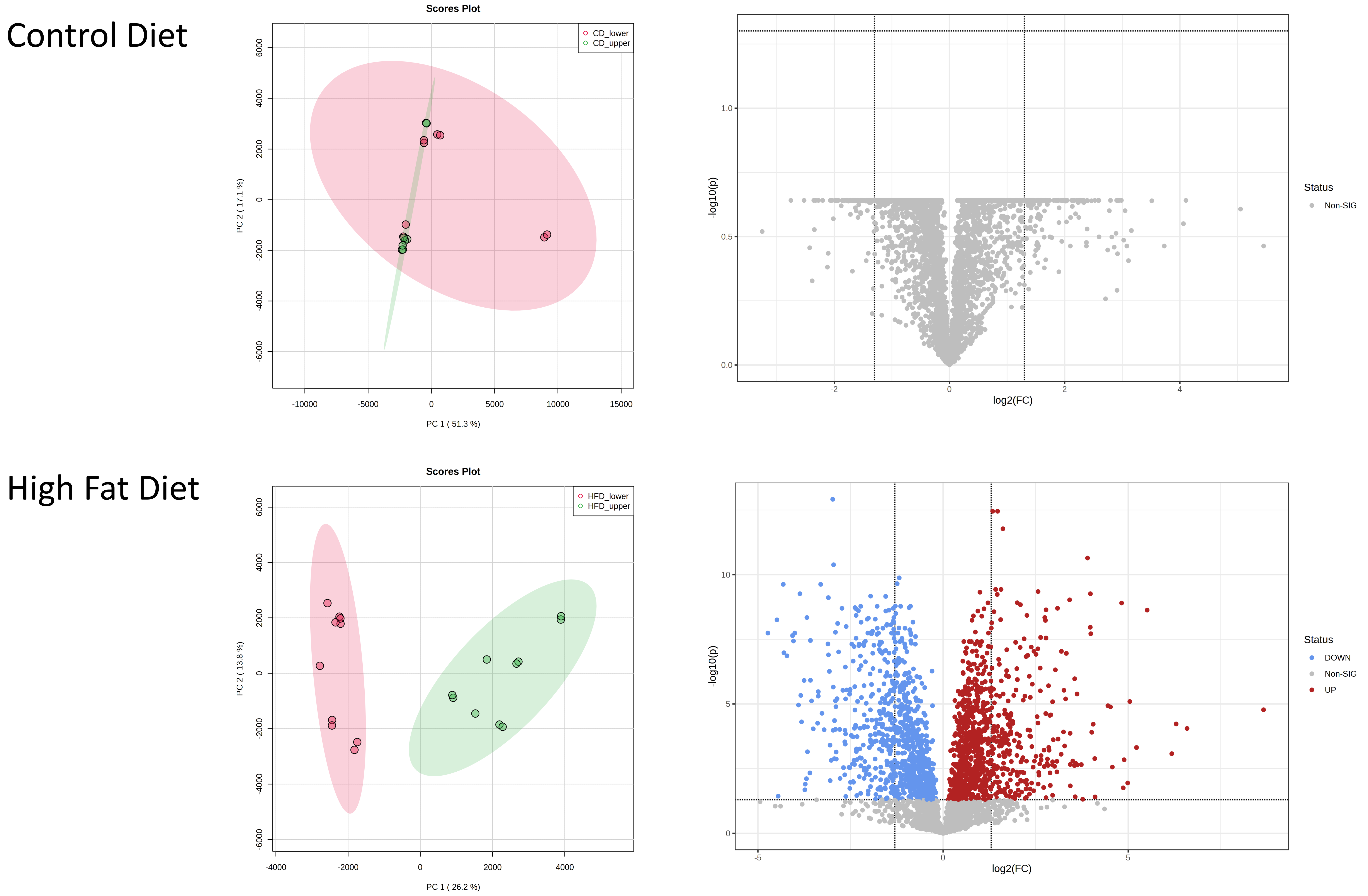

Figure S1. High-throughput deep proteomics of intestinal epithelial cells (IECs) in the upper and lower small intestine of mice fed control (CD) or high-fat diet (HFD). Principal component analysis of the proteome of IECs shows that in the HFD group there is a large difference in the global pattern of protein expression in the upper and lower small intestine. Volcano plots of identified 854 differentially expressed proteins (DEPs) in the HFD group while no DEPs were found in the upper and lower IECs in the CD group. Red and blue dots indicate proteins with increased and decreased expression in the IECs of the lower small intestine.

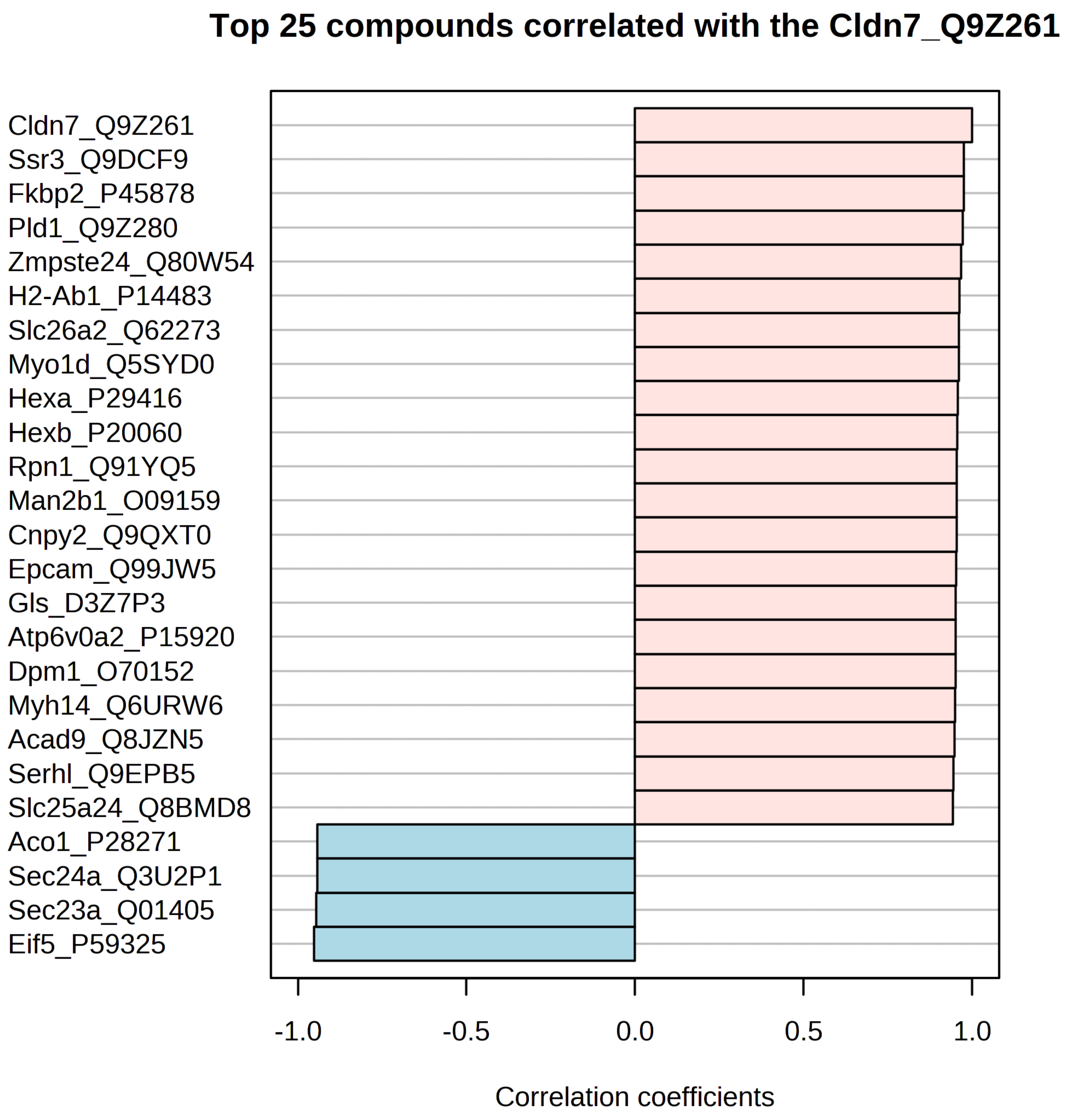

Figure S2. Top 25 proteins with strong positive or negative correlation to Cldn7 and expression levels by quantitative proteomic analysis.
